# Low back pain should be considered a health and research priority in Brazil: Lost productivity and healthcare costs between 2012 to 2016

**DOI:** 10.1371/journal.pone.0230902

**Published:** 2020-04-01

**Authors:** Rodrigo Luiz Carregaro, Caroline Ribeiro Tottoli, Daniela da Silva Rodrigues, Judith E. Bosmans, Everton Nunes da Silva, Maurits van Tulder

**Affiliations:** 1 School of Physical Therapy, Master in Rehabilitation Sciences, Universidade de Brasília (UnB), Campus UnB Ceilândia, Brasília, Brazil; 2 Núcleo de Evidências e Tecnologias em Saúde (NETecS), Universidade de Brasília (UnB), Campus UnB Ceilândia, Brasília, Brazil; 3 School of Occupational Therapy, Universidade de Brasília (UnB), Campus UnB Ceilândia, Brasília, Brazil; 4 Department of Health Sciences, Faculty of Science, Vrije Universiteit Amsterdam, Amsterdam, The Netherlands; 5 School of Collective Health, Universidade de Brasília (UnB), Campus UnB Ceilândia, Brasília, Brazil; 6 Department of Physiotherapy & Occupational Therapy, Aarhus University Hospital, Aarhus, Denmark; Universita degli Studi di Napoli Federico II, ITALY

## Abstract

**Background:**

Low Back Pain (LBP) is associated with an increase in disability-adjusted life years, and increased risk of disability retirement and greater absenteeism in Brazil. Hence, evidence on healthcare and lost productivity costs due to LBP is of utmost importance to inform decision-makers.

**Methods:**

Cost-of-illness study with top-down approach, and societal perspective. We extracted data from National databases, considering the period 2012–2016. Outpatient expenses included clinical, surgical, diagnosis, orthosis/prosthetics, and complementary actions. Inpatient care expenses included hospital and professional services, intensive care unit, and companion stay. For productivity losses, duration of work absence and associated information (work-related and non-work-related; value of the sickness absence benefit; age; gender; and economic activity) were analyzed. Lost productivity costs were calculated multiplying the absence from work (days) by the daily-benefit.

**Results:**

The societal costs amounted to US$ 2.2 billion, and productivity losses represented 79% of the costs. Total healthcare expenses were estimated to US$ 460 million. We found more than 880,000 diagnostic images. Individuals with LBP were in total 59 million days absent from work between 2012–2016. The mean lost days absent from work per person, for each year investigated was, respectively, 88; 84; 83; 87; and 100. Men were more days absent from work than women. In addition, rural workers presented greater absence from work compared to other professional activities.

**Conclusion:**

Healthcare expenses and lost productivity costs due to LBP were substantial, hence, there is a need for improvement of health services and policies to deal with this increasing burden of illness. We found an extensive use of diagnostic imaging, which is rather discouraged by clinical guidelines. We assume that men were experiencing high levels of back pain disability compared with women, as they presented greater absenteeism and higher lost productivity costs.

## Introduction

Low Back Pain (LBP) is a prevalent, incapacitating and challenging condition [1, 2]. Currently, LBP is considered one of the most disabling chronic conditions worldwide [3, 4]. Previous studies in several countries have reported that LBP is associated with an increase in disability-adjusted life years (DALYs), which impacts the worker’s individual productive capacity resulting in productivity losses due to absenteeism [3–7]. Productivity losses are associated with production loss and replacement costs due to illness, disability and death of productive individuals [8, 9]. Patients with LBP are high users of healthcare services, including rehabilitation services, and have high productivity losses due to this condition [10]. Hence, there is a burden to healthcare systems and society [11].

Information on healthcare costs and productivity losses due to chronic diseases is useful to inform policy makers and to guide preventive and rehabilitation strategies [12]. Recently, researchers have highlighted the importance of monitoring and understanding the healthcare spending associated with LBP in order to optimize the use of scarce resources and to adopt evidence-based interventions [13, 14]. This is essential as a basis for the development of future cost-saving cost-effective intervention strategies [1, 15].

There is a growing prevalence of LBP in Brazil [16], resulting in an increase of 79% in the total number of years lived with disability between 1990 and 2016 [17]. Previous findings showed a LBP prevalence ranging from 10% to 50% in different populations, such as adolescents, adults, and elderly [18–22]. Thus, a large share of the economic active population might be experiencing disability and functional limitations as a consequence of LBP, which leads to negative socioeconomic impacts [23, 24]. In a previous study [25], we demonstrated that in 2016 the Brazilian government spent US$ 71.4 million on direct healthcare expenses associated with spinal disorders, with LBP alone accounting for approximately 67% of the costs. Furthermore, LBP was associated with an increased risk of disability retirement and greater absenteeism among Brazilian health system users [5]. However, the previous studies did not investigate the lost productivity costs exclusively due to LBP.

There is scarce evidence regarding productivity losses related to LBP in Brazil. Although previous studies have shown a large number of sickness absence claims [23, 24, 26, 27], to the best of our knowledge, the investigation of productivity losses influenced by LBP remains to be unraveled. Therefore, the aim of the present study is to estimate the healthcare expenditure and productivity losses related to LBP incurred within the Brazilian Public Healthcare System and the National Institute of Social Security, between 2012 and 2016. The secondary aims were to estimate if healthcare costs and productivity losses differ between male and female individuals; to estimate if socioeconomic factors and gender might be associated with a greater absence from work and to higher lost productivity costs.

## Method

### Study design

This is a cost-of-illness study with a prevalence-based design (i.e., measures the impact of prevalent conditions), and a top-down approach (i.e., population-based method in which national healthcare databases are consulted) from a societal perspective.[28]

The Institutional Ethics Committee granted approval for this study (Faculdade de Ceilândia, protocol n. 1.969.372, 16/03/2017).

### Population

We included information from adults (aged from 19 years and older). For analysis purposes, we stratified individuals into the following age groups: 19-28y; 29-38y; 39-48y; 49-58y; 59-68y; 69-78y; >79 years. We used official government information from the Brazilian Institute of Geography and Statistics (IBGE), containing the population of each age group, considering the period of 2012 to 2016.

### Data sources

Data were collected from National databases, considering the period between 2012 until 2016. The following ICD-10 codes were used to define the presence of LBP: M40.4 (Other lordosis); M40.5 (Lordosis, unspecified); M51 (Other intervertebral disc disorders); M54.1 (Radiculopathy); M54.3 (Sciatica); M54.4 (Lumbago with sciatica); M54.5 (Low back pain); M54.8 (Other dorsalgia); and M54.9 (Dorsalgia, unspecified).

To estimate the expenses of healthcare utilization associated with LBP, we extracted data from the Brazilian Health Ministry’s Hospital Information System (SIH; inpatient care) and Outpatient Information System (SIA; outpatient care). In order to estimate productivity losses, data from the Ministry of Social Insurance (INSS) was used, and included information regarding the duration of the work absence due to disabilities and associated information (category of benefit—work-related and non-work related; value of the monthly sickness absence benefit; age; gender; economic activity; and geographical location).

### Inpatient and outpatient care expenses

The Hospital Information System contains all records of inpatient care, which are processed and sent to the Ministry of Health and included in a National Database. The Outpatient Information System includes all outpatient care by public and private providers contracted by the Brazilian Public Healthcare System (SUS). Individual patient data were not available because expenses are aggregated and linked with the inpatient or outpatient admission document number, not with the individual name or identity number. Consequently, individual analyses were not possible, because there may be situations in which there is more than one procedure recorded in the same admission document, and there may be more than one admission document for the same person.

The expenses are based on reimbursement values determined by the Brazilian Ministry of Health, that is the payments that are made to healthcare providers who deliver care in the public health system setting. For inpatient care, the following cost items were obtained: hospital services (i.e., daily rate; room fees; food; personal hygiene; bed support; hospital supplies; allied healthcare professional services; medications and diagnostic and therapeutic auxiliary services); professional services (health professionals); intensive care unit (ICU) expenses (including the use of all equipment for intensive care, technical teams, and 24-hour patient monitoring); companion daily stay (the Brazilian regulations allow, for each patient, one companion during the hospitalization, and includes adequate accommodation and provision of the main meals).

Outpatient care expenses included ambulatory services/procedures, such as medical and allied healthcare consultations, examinations, diagnostic imaging, clinical and surgical procedures, physiotherapy procedures, and other procedures.

### Productivity losses

Workers from formal labor market are covered by social security, which is managed by the National Institute of Social Security (INSS) [29], representing approximately one-third of the worker’s population [30]. Temporary or permanent social security benefits are paid to taxpayers who are unable to work due to illness after being evaluated by a physician from the INSS. In Brazil, disability benefits characterized by sick leave are classified into two types: (1) Work-related sickness benefit: granted to insured persons incapacitated by occupational and commuting accidents, and occupational diseases; (2) non-work-related benefit: social security benefit granted to insured persons disabled by illness or injury of any kind. The first fifteen days of absence are paid by the employer and, after approval of sickness absence by the expert physician, the INSS is responsible for paying the benefits [26].

We performed a calculation considering the monthly-base period by dividing the monthly benefit by 22 days, considering 40 hours per week excluding Saturdays and Sundays, to estimate the daily value of the benefit. Subsequently, the lost productivity costs (in US$) were calculated using the Human Capital Approach, by multiplying the total amount of absence from work (in days) by the daily benefit (work-related and non-work-related, for each year– 2012 until 2016). For the lost productivity costs, individual data were available.

### Data analysis

Data were presented descriptively, estimating the mean and standard deviation of variables used in the analysis. The Tabwin software version 4.1.5 was used for data extraction and processing. The outpatient expenses were reported separately for the following categories: clinical, surgical, diagnosis, orthosis and prosthetics, and complementary actions. Inpatient care expenses were presented separately for hospital and professional services, ICU, and companion stay. As all expenses are aggregated within the hospital system, the discrimination of each category was not possible.

Cost data (healthcare and lost productivity) were extracted in Brazilian *Reais* (R$) and adjusted to the year 2016 using Brazilian consumer price indices (IPCA). Next, costs were converted to American Dollars (US$) based on information from the World Bank Power Purchase Parities—PPP [31]. The PPP for Brazil in 2016 was of 1.983.

Considering individual data on productivity losses, we adopted a generalized linear model [32] with a gamma regression to the response variables absence from work (in days), and lost productivity costs per person (in US$), to determine which factors contribute the most to a greater absence from work and lost productivity costs. This model was chosen because allows non-normal distributions (e.g., skewed cost data), and provide inferences about the mean costs directly, which is appropriate for health economics investigations [32]. Explanatory variables were gender (male; female), economic activity (commerce; transports; industry; public servant; rural work), type of benefit (work-related and non-work related), and age (included as a covariate). Regarding economic activities, commerce was considered the reference, because this category presented the highest number of sickness absence claims in Brazil [23]. Only explanatory variables which contributed significantly to the model (*p*<0.05) were included. For the model estimation, we adopted the identity link function. Goodness of fit was verified by the AIC (Akaike Information Criterion). The correlation matrix was analyzed and variables that were highly correlated (r > 0.7) were considered collinear.

Significance was set at 5% (*p*<0.05). Statistical analysis was performed in SPSS version 25.

## Results

In the 5-year period investigated (2012–2016), total societal costs amounted to US$ 2.2 billion for LBP in Brazil, and productivity losses represented 79% of these costs (Table 1).

**Table 1:** Overview of the healthcare expenditures and productivity losses attributable to Low Back Pain (LBP) in Brazil, from 2012 to 2016, percentages of costs were referenced (%) to the total amount (direct plus indirect costs), for each year.

Components	2012		2013		2014		2015		2016		Total 2012–2016
		%		%		%		%		%	
**Healthcare expenses**											
Inpatient Care (in US$):											
Hospital Services	50,208,543	10.9	55,325,724	11.9	55,503,601	11.8	39,486,153	10.6	32,937,233	6.9	233,461,254
Professional Services	8,351,664	1.8	8,655,584	1.9	8,343,792	1.8	6,306,466	1.7	5,511,082	1.2	37,168,588
ICU	1,741,554	0.4	1,815,920	0.4	2,069,947	0.4	1,460,922	0.4	1,319,926	0.3	8,408,269
Companion Stay	164,071	<0.1	179,188	<0.1	194,470	<0.1	173,731	<0.1	162,113	<0.1	873,573
*Total Inpatient*	*60,465,832*	*13.1*	*65,976,416*	*14.2*	*66,111,810*	*14.1*	*47,427,272*	*12.7*	*39,930,354*	*8.4*	*279,911,684*
Outpatient Care (in US$):											
Diagnostic Procedures	22,573,833	4.9	20,250,153	4.3	18,838,016	4.0	16,886,794	4.5	16,024,856	3.4	94,573,652
Clinical Services	21,648,258	4.7	19,351,600	4.2	16,518,009	3.5	14,732,768	4.0	14,372,056	3.0	86,622,691
Surgery	31,941	<0.1	32,444	<0.1	21,368	<0.1	12,013	0.0	11,892	<0.1	109,658
Orthoses and prostheses	363,741	0.1	324,739	0.1	294,221	0.1	216,615	0.1	219,126	<0.1	1,418,441
Complementary actions	108,789	<0.1	72,525	<0.1	58,636	<0.1	54,213	0.0	33,345	<0.1	327,507
*Total Outpatient*	*44,726,562*	*9.7*	*40,031,461*	*8.6*	*35,730,250*	*7.6*	*31,902,402*	*8.6*	*30,661,275*	*6.4*	*183,051,951*
***Total healthcare costs***	***105,192,394***	***22.8***	***106,007,877***	***22.8***	***101,842,060***	***21.7***	***79,329,674***	***21.3***	***70,591,629***	***14.8***	***462,963,635***
**Productivity losses**											
Lost days off work:											
Mean (SD)	88.0 (62.5)		83.9 (56.0)		83.3 (53.9)		87.1 (57.1)		99.9 (73.4)		
Sum	11,751,650		11,771,427		11,977,032		9,870,608		13,741,707		***59*,*112*,*424***
*Lost productivity costs (in US$)*	*355,539,135*	*77.2*	*359,887,195*	*77.2*	*367,714,625*	*78.3*	*293,349,002*	*78.7*	*407,481,561*	*85.2*	***1,783,971,518***
***Total societal costs (in US$)***	***460,731,529***	***100.0***	***465,895,072***	***100.0***	***469,556,686***	***100.0***	***372,678,676***	***100.0***	***478,073,190***	***100.0***	***2,246,935,153***

ICU: Intensive care unit; SD: Standard deviation.

**Fig 1:**
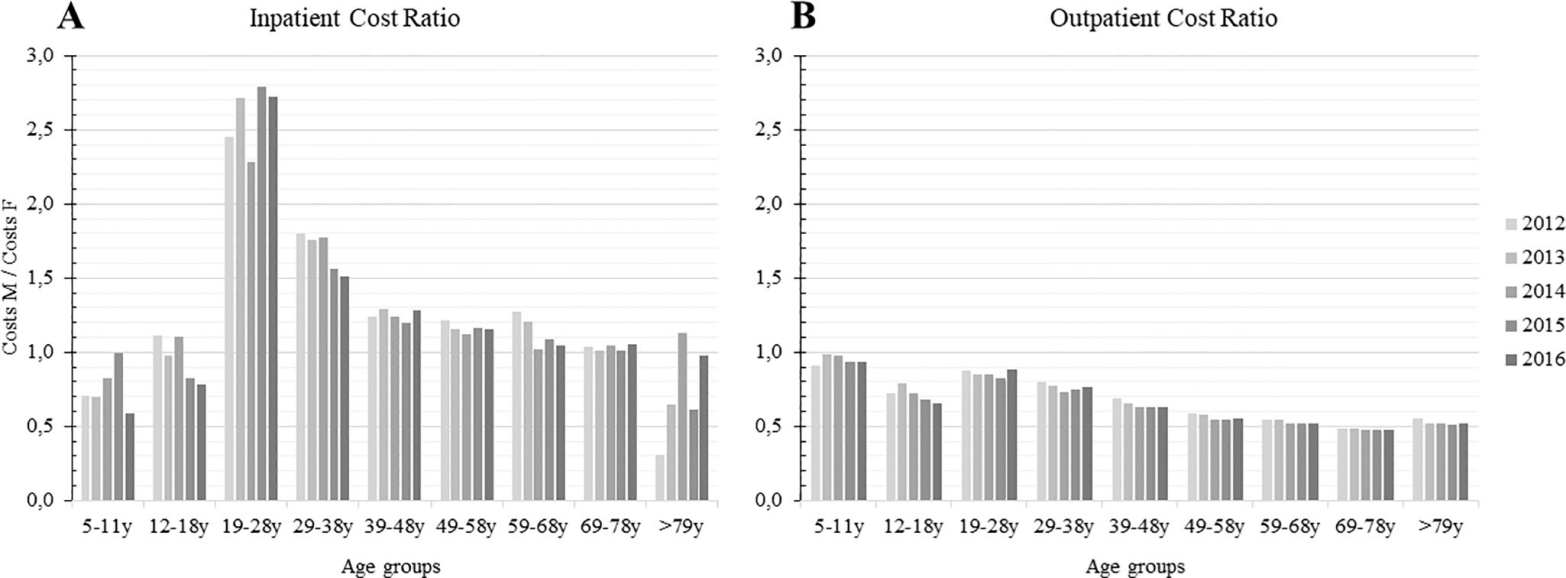
Ratio of the treatment expenses with low back pain (LBP) in Brazil (2012–2016), between male (M) and female (F) individuals (A: Inpatient care; B: Outpatient services).

Total healthcare expenses were estimated to approximately US$ 460 million in the 5-year period investigated (2012–2016). The number of inpatient admissions due to LBP in this period was 118,705. Individuals between 39 to 48 and 49 to 58 years of age presented the highest number of inpatient duration days, in each year. Details regarding the duration of inpatient admission, and costs among men and women are presented in Appendix S1 Table. Hospital expenses were 2.5 times higher for males aged between 19–28 years, and 1.5 times higher between 29–38 years, compared to female individuals within the same age groups. Outpatient care expenses were 1.5 times higher for females aged between 49–78 years, compared with males (Fig 1).

Use of diagnostic imaging is presented in Table 2. More than 80% of all diagnostic procedures were characterized by the use of MRI and CT-scans (Appendix S2 Table). Our findings demonstrated that more than 886,000 diagnostic imaging procedures were performed between 2012–2016, including around 442,000 CT-scans (49.8%) and 436,000 Magnetic Resonance Imaging (49.2%). On average, around 10.6 images per 1,000 individuals were performed (Table 2). Clinical and diagnostic procedures as part of outpatient care accounted for the major share of outpatient expenses. Physiotherapy interventions was the most commonly implemented intervention (around 6 million sessions each year).

**Table 2:** Diagnostic imaging use during inpatient and outpatient care of individuals with Low Back Pain (LBP) in Brazil (2012–2016).

	2012	2013	2014	2015	2016	Total
**Outpatient care:**						
MRI	84,141	82,137	85,636	87,495	83,826	423,235
CT-Scan	91,707	83,913	86,461	77,002	73,172	412,255
*Total outpatient*	*175*,*848*	*166*,*050*	*172*,*097*	*164*,*497*	*156*,*998*	*835*,*490*
**Inpatient care:**						
MRI	726	2,347	2,991	3,516	3,440	13,020
CT-Scan	384	7,026	7,925	7,348	7,145	29,828
Radiography	32	84	89	53	47	305
Ultrasound	2,036	1,332	1,519	1,454	1,539	7,880
*Total inpatient*	*3*,*178*	*10*,*789*	*12*,*524*	*12*,*371*	*12*,*171*	*51*,*033*
***Total***	***179*,*026***	***176*,*839***	***184*,*621***	***176*,*868***	***169*,*169***	***886*,*523***
Number of diagnostic imaging/1,000 individuals with LBP*	11.01	10.73	11.05	10.43	9.86	**10.61**

MRI: Magnetic Resonance Image; CT-Scan: Computerized Tomography.

*Based on the Global Burden of Disease Collaborative Network findings.

Regarding lost productivity costs, our estimates were based on data from 668,206 individuals with sickness absence due to LBP, who were insured by the INSS. For Brazil, patients with LBP were in total 59 million days off work due to LBP between 2012–2016 (Table 1). The mean absenteeism days from work per person, for each year investigated was, respectively, 88 ± 62 (2012); 84 ± 56 (2013); 83 ± 54 (2014); 87 ± 57 (2015); and 100 ± 73 (2016). Data on sick leave is presented in Table 3. Overall, we found that more men were absent from work, and they had more absenteeism days compared to women.

**Table 3:** Data on sick leave due to low back pain in the 5-year period investigated, for male and female workers.

	2012	2013	2014	2015	2016
Male					
N	78,721	80,418	80,424	63,327	76,834
Age (years–$\overset{-}{X}$; SD)	44.0 (10.2)	44.4 (10.3)	44.5 (10.3)	44.8 (10.3)	45.1 (10.3)
Absenteeism days ($\overset{-}{X}$; SD)	88.6 (63.2)	85.3 (67.1)	85.5 (54.7)	89.4 (58.1)	102.3 (73.1)
Monthly benefit (in US$—$\overset{-}{X}$; SD)	562.9 (317.3)	606.2 (335.3)	645.5 (351.0)	694.4 (375.4)	744.2 (403.7)
Female					
N	54,724	59,927	63,267	49,954	60,610
Age (years–$\overset{-}{X}$; SD)	44.4 (10.1)	44.5 (10.2)	44.4 (10.4)	44.8 (10.3)	45.1 (10.3)
Absenteeism days ($\overset{-}{X}$; SD)	87.2 (61.5)	82.1 (54.6)	80.5 (52.6)	84.2 (55.4)	96.7 (72.1)
Monthly benefit (in US$—$\overset{-}{X}$; SD)	421.5 (233.2)	455.7 (244.4)	488.5 (255.6)	528.2 (274.9)	572.3 (209.3)

$\overset{-}{X}$: Mean; SD: Standard Deviation.

The results of the regression analysis are presented in Table 4, and the descriptive results are presented in Appendix S3 Table. We did not identify any collinearity between variables.

**Table 4:** Regression analysis on absence from work (in days), and lost productivity costs (in US$), considering the predictors gender (male; female), economic activity (commerce; transports; industry; public servant; rural work), type of benefit (work-related and non-work-related sickness benefit), and age (in years; included as a covariate).

Absence from work (in days)	B (SE)	95%CI	*p-value*	Lost productivity costs (in US$)	B (SE)	95%CI	*p-value*
Intercept	50.49 (0.36)	49.79; 51.19	-	Intercept	1186.9 (12.9)	1161.7; 1212.1	-
Gender†	4.06 (0.14)	3.77; 4.34	<0.001	Gender†	662.3 (5.1)	652.2; 672.4	<0.001
Type of benefit*	-2.07 (0.22)	-2.50; -1.65	<0.001	Type of benefit*	41.1 (7.5)	26.3; 55.8	<0.001
Economic activity:				Economic activity:			
Transports	-1.43 (1.22)	-3.83; 0.96	0.24	Transports	1305.9 (76.2)	1156.5; 1455.5	<0.001
Public servant	-6.41 (5.63)	-17.47; 4.63	0.25	Public servant	-186.9 (187.6)	-554.8; 180.9	0.31
Rural work	23.14 (0.28)	22.59; 23.68	<0.001	Rural work	-429.3 (6.7)	-442.4; -416.1	<0.001
Industry	-16.26 (7.95)	-31.85; -0.67	0.04	Industry	-431.1 (313.4)	-1045.4; 183.1	0.16
Commerce^a^	-	-	-	Commerce^a^	-	-	-
Age	0.78 (0.006)	0.76; 0.79	<0.001	Age	17.1 (0.2)	16.6; 17.6	<0.001

SE: Standard Error

^†^reference category is female gender;

*reference category is work-related sickness benefit;

^a^ redundant parameter because it is the reference.

Akaike Information Criterion (AIC) for absence from work: 7315299.8; AIC for Lost productivity costs: 11670245.5

Regarding absence from work, the findings showed that men presented more absenteeism days than women. Rural workers presented greater absence from work, and industry activities less absence from work, compared to the other professional activities. In addition, both age and type of benefit contributed significantly to the model, though the difference between work-related sickness benefits and non-work-related was small (around 2 days).

We found a mean lost productivity cost per individual of US$ 2,684.6 (CI95%: 2,538.2; 2,831.1) for male individuals, and US$ 2,022.3 (CI95%: 1,875.8; 2,168.8) for female individuals (Appendix S3 Table). Rural workers had a significantly lower lost productivity cost; and transports activities presented a higher lost productivity cost, when compared to the other professional categories. Both age and type of benefit contributed significantly to the regression model (Table 4).

## Discussion

The aim of the present study was to estimate the healthcare and lost productivity costs due to LBP in Brazil. We showed that between 2012–2016, US$ 2.2 billion was spent on LBP, with the largest share spent on productivity losses. In addition, men presented more absenteeism from work compared with women. We found that rural work was associated with greater absenteeism and industry activities with less absenteeism, though transports and commerce activities showed the highest lost productivity costs. Increases in age were significantly associated with longer absenteeism and higher lost productivity costs.

We found a total expense of around US$ 2.2 billion for patients with LBP in Brazil (annual expending of around US$ 500 million). This finding is consistent with previous research from other countries, showing high healthcare and lost productivity costs among individuals with LBP [6, 10, 15, 33, 34]. For instance, the lost productivity costs related to disability due to chronic spine pain in Portugal in 2010 was approximately € 739 million, mostly in individuals aged between 50 to 59 years [7]. Additionally, in 2011, the mean cost per episode/patient with LBP in Sweden was estimated in € 2,753; totaling € 739 million annually, and 66% were attributed to lost productivity costs [15]. In addition, the trend from 2012 to 2016 showed slight reductions in healthcare costs but increases in lost productivity costs. These findings show that although Brazil is considered an upper middle-income country, the impacts of LBP are similar to those from high-income and more industrialized ones, though some countries implemented successful disability policies to reduce productivity losses [10]. A previous study demonstrated that individuals with chronic pain in emerging economies, including Brazil, have the same level of absenteeism compared to developed countries, but lower physical and mental health-related quality of life [35]. This is relevant, because high-income countries have stronger policies and widespread investment to support preventive strategies aiming to reduce or minimize the disability due to LBP [10].

We have shown that between 2012 and 2016, total inpatient care costs among men were higher than among women, although outpatient care costs were higher for women. We demonstrated similar findings in a study estimating healthcare expenses associated with spinal disorders [25]. Further studies demonstrated that women were more affected by LBP [22, 35, 36], thus resulting in higher healthcare and lost productivity costs [7]. However, we found that Brazilian male workers seems to be more affected by LBP. This finding might be explained by the healthcare-seeking behavior of men, which is influenced by masculinity, self-reliance, and patriarchal characteristics [37]. Usually, men use health services less often compared with women [37, 38], and they may also ignore or delay healthcare due to illness [38]. Contrarily, women tend to use health services more frequently, mostly for routine screening, and prevention [39, 40]. A previous study investigated different professional activities and demonstrated that work-related injuries are more prevalent in male workers, and although women presented a higher incidence of sickness absence, the difference was small (less than 4%) [26]. Previous findings showed higher exposures to occupational risk factors on male workers due to higher rates in the labor force [41]. Additionally, estimates of disability-adjusted life years due to LBP were 50% higher for men compared with women [41]. Therefore, we hypothesize that men were experiencing high levels of back pain disability as a result of the delay in seeking health services, and work-related injuries.

We found more than 880,000 routine diagnostic imaging procedures were performed between 2012–2016 during inpatient and outpatient care, with a ratio of approximate 10 images per 1000 individuals affected by LBP in Brazil. Inappropriate use of routine diagnostic imaging for LBP is being widely discussed [14, 42–45]. Previous studies demonstrated frequent referral for diagnostic imaging, despite the absence of red flags, such as infection, progressive neurologic deficits, or underlying pathologies [44, 46]. However, current recommendations rather discourage imaging as part of early management of LBP, because of harms such as unnecessary exposition to radiation, patient fear and sense of fragility, and use of additional procedures resulting in overmedicalization [14, 44]. Nonetheless, a systematic review [45] found a 53% relative increase in diagnostic imaging use (e.g., CT-scans) for LBP in primary and emergency care, from 1995 to 2015, despite guidelines advice and campaigns otherwise [47]. Clinicians and decision-makers should be aware that unnecessary diagnostic imaging wastes scarce financial resources [45, 48], and has little effect on clinical outcomes [47, 48]. This finding warrants further research aiming to design specific strategies and interventions to reduce unnecessary diagnostic imaging in the Brazilian health system. We would recommend further studies to understand the clinical-decision reasoning and aspects that might explain the overuse of diagnostic imaging for LBP in Brazil [45, 49], to improve evidence-based decision support [50].

Physiotherapy sessions were extensively employed, mostly during outpatient care. This is a compelling finding because several international clinical guidelines [47, 51] recommends conventional interventions widely adopted by physiotherapists, such as exercise and manual therapy, for the improvement of clinical outcomes and health-related quality of life. For instance, exercise interventions (e.g., strength, flexibility, core training) significantly improved physical function in individuals with chronic pain, including those with LBP [52]. Also, a systematic review demonstrated that individually designed strengthening or stabilizing exercise programs were considered effective in healthcare settings, with clinically significant improvements on pain intensity [53]. These findings are applicable to the decision-making of Brazilian health system managers, that might consider expanding the use of physiotherapy interventions (combined or not with health education) as routine use in patients with chronic LBP [14]. Notwithstanding, these findings must be interpreted with caution as we did not have access to the specific routines adopted by Physiotherapists. Moreover, we would recommend further investigations on whether professionals are providing care according to treatment guidelines and evidence-based decisions.

The mean days of absence from work were around 80 to 100 days in each year investigated, and the total costs related to absence from work were around 59 million days. Male workers had a greater sickness absence benefit and a longer absence from work compared with women. Furthermore, rural workers presented the longest time off work, however, with the lowest sickness absence benefit. We assumed that more than 80-days off work is high and impacting to work productivity and quality of life. This is in line with a systematic review [54] demonstrating that workers who have not returned to work after 30-days were deemed eligible for interventions to prevent longer absences, higher costs, and adverse socioeconomic consequences. Other studies showed that occupational risk factors such as excessive workload, need for rest and poor health perception were associated with a longer absence from work in professional drivers [55], and industry activities [23]. A study investigating the perceptions of farm workers in New Zealand regarding presenteeism despite having LBP [56], showed that although farmers are fully aware of the occupational risks, they are driven by economic reasons and resilience to stay active and complete the tasks. We may extrapolate these findings to Brazil, as the physical exertion is not likely to differ from farm workers from other countries. In fact, previous studies in Brazil investigated rural workers, and demonstrated that manual lifting and heavy tasks were limiting factors to occupational activities, and LBP also interfered with daily activities [57, 58]. Thus, it is probable that rural workers requested sickness benefits in a worsened clinical condition, which might explain a longer absence from work and healthcare costs.

### Implications for policy makers

Even though there are effective strategies of health surveillance in Brazil to monitor the prevalence and incidence of chronic disorders such as LBP, there is a need for improvement of health services and policies to deal with this increasing burden of illness [59]. Hence, our findings demonstrate that LBP should be considered a health and research priority in Brazil, though a challenge would be to ensure the rational and strategic allocation of financial resources according to priorities in each state and region [17]. We suggest that the employment of clinical protocols and guidelines is important to ensure the sustainability of the public health system as well as the National Insurer. For instance, adherence of patients with LBP to a Dutch physiotherapy clinical guideline was associated with improvements in physical functioning, and fewer treatment sessions, indicating a better treatment efficiency [60]. Moreover, patients with LBP receiving early referral to physiotherapists had a decreased likelihood of further diagnostic imaging, additional physician visits, surgery, lumbar spine injections and medication [61]. Likewise, subsequent total healthcare costs were lower to those patients receiving early physiotherapy [61].

We raise the importance of prevention and health promotion, in order to improve the worker’s health and coping strategies, aiming to the reduction of sickness absence and costs. This is of utmost importance, as a previous study demonstrated that increased life satisfaction, despite having LBP, social support at work, and job satisfaction, were significant predictors of less work absence [12]. In addition, a cohort study [34] investigated the care pathway of patients with LBP and demonstrated that the adoption of physiotherapy interventions and a higher health-related quality of life were significantly associated with reduced healthcare costs.

### Study limitations

Our findings might have been influenced by the poor and/or inaccurate registration of health systems, and wide variability in the adoption of ICD-10 codes as primary diagnosis.

In the period investigated, the total expenses of inpatient and outpatient care in Brazil was US$ 82.6 billion [62], and the Gross Domestic Product of Brazil in 2016 was US$ 1.796 Trillion [63]. These data put the expenses of LBP into perspective, as they represented 0.2% of total healthcare expenses and less than 0.1% of the Brazilian GDP. Although the expenses with LBP are lower compared to other conditions, such as obesity [64], it is highly prevalent and incapacitating [17].

The database adopted for calculating productivity losses are exclusively related to absenteeism. Hence, we were not able to include estimates of lost productivity costs due to presenteeism. Moreover, we investigated the vast majority of private-sector workers but few of the public-sector (we found less than 0.1% of public civil servants). Usually, public-sector workers have a separate system for sickness benefit claims, but there is no database registry available. Approximately 54 million individuals are formally included in the private-sector, and there were estimates indicating a total number of 11.2 million public-sector workers in 2016 [65]. By applying the same proportion of workers from the private sector that were on sickness leave due to LBP in the present study (which was roughly 1.2% of the total number of private-sector workers in 2016), we estimated that approximately 134 thousand public-sector workers would have been absent from work in the same period. The average wage for public-sector workers in Brazil in 2016 was approximately US$ 2,000 (daily-wage of US$ 90) [65]. Hence, assuming the same amount of days absent from work (mean of around 85 days), we estimate that the indirect cost for public-sector workers in Brazil would add up about US$ 1.02 billion. Therefore, actual LBP expenses in Brazil may be even higher.

## Conclusion

We found that healthcare expenses and productivity losses due to LBP in Brazil between 2012–2016 were substantial, and lost productivity costs represented of the largest share. We found an extensive use of diagnostic imaging, mostly MRI and CT-scans. We demonstrated that men had more absenteeism days and higher lost productivity costs compared to women.

## Supporting information

S1 Table Data on the duration of inpatient admissions (in days), and costs per hospital admission among men and women stratified by age groups. (DOCX)

S2 Table Overview of clinical and diagnostic procedures/services adopted during outpatient care of individuals with low back pain between 2012–2016. Q%: Percentage relative to the total number of procedures reported during outpatient care in each year; C%: Percentage relative to the total costs in each year.
(DOCX)

S3 Table Estimated marginal means on absence from work (in days), and indirect costs (in US$), stratified by gender (male; female), economic activity (commerce; transports; industry; public servant; rural work), and type of benefit (Work-related and non-work-related benefit). SE: Standard Error; 95%CI: 95% confidence interval.
(DOCX)
