# Supplementary material for: Low back pain should be considered a health and research priority in Brazil: Lost productivity and healthcare costs between 2012 to 2016
Source: PLoS One. 2020 Apr 1;15(4):e0230902. doi: 10.1371/journal.pone.0230902 (PMC7112211; doi:10.1371/journal.pone.0230902)
Supplement: S1 Table — (DOCX) [file pone.0230902.s001.docx]

**S1 Table. Data on the duration of inpatient admissions (in days), and costs per hospital admission among men and women stratified by age groups.**

|  | | **Age Groups (years)** | | | | | | |
| --- | --- | --- | --- | --- | --- | --- | --- | --- |
| **2012** | | **19-28** | **29-38** | **39-48** | **49-58** | **59-68** | **69-78** | **>79** |
| Inpatient duration (in days) | Men | 7927 | 14156 | 20507 | 20441 | 12908 | 5072 | 1106 |
|  | Women | 4324 | 10117 | 17502 | 16341 | 10494 | 5585 | 1568 |
| Cost/hospital admission (in US$) | Men | 3302 | 2604 | 2825 | 3026 | 3544 | 2837 | 1513 |
|  | Women | 1926 | 1846 | 2376 | 2604 | 2744 | 2107 | 2938 |
| **2013** | | **19-28** | **29-38** | **39-48** | **49-58** | **59-68** | **69-78** | **>79** |
| Inpatient duration (in days) | Men | 8052 | 14698 | 20853 | 20922 | 13642 | 5932 | 942 |
|  | Women | 4047 | 9282 | 16583 | 17228 | 11255 | 6377 | 1770 |
| Cost/hospital admission (in US$) | Men | 3495 | 2581 | 2932 | 3305 | 3422 | 2897 | 1305 |
|  | Women | 2044 | 1844 | 2496 | 2813 | 2826 | 2330 | 1002 |
| **2014** | | **19-28** | **29-38** | **39-48** | **49-58** | **59-68** | **69-78** | **>79** |
| Inpatient duration (in days) | Men | 7287 | 15470 | 20738 | 20805 | 13601 | 6514 | 1747 |
|  | Women | 4088 | 10463 | 17039 | 19006 | 12520 | 6807 | 2172 |
| Cost/hospital admission (in US$) | Men | 2973 | 2592 | 2739 | 3111 | 3223 | 2955 | 1781 |
|  | Women | 1856 | 1898 | 2317 | 2719 | 2927 | 2258 | 1051 |
| **2015** | | **19-28** | **29-38** | **39-48** | **49-58** | **59-68** | **69-78** | **>79** |
| Inpatient duration (in days) | Men | 5611 | 12543 | 18459 | 19334 | 13721 | 6150 | 1129 |
|  | Women | 3187 | 10817 | 15850 | 16592 | 12180 | 6249 | 1914 |
| Cost/hospital admission (in US$) | Men | 1818 | 1619 | 1933 | 2354 | 2650 | 2185 | 639 |
|  | Women | 928 | 1346 | 1691 | 1982 | 2253 | 1722 | 692 |
| **2016** | | **19-28** | **29-38** | **39-48** | **49-58** | **59-68** | **69-78** | **>79** |
| Inpatient duration (in days) | Men | 5346 | 12084 | 17797 | 17312 | 13244 | 5332 | 1625 |
|  | Women | 3747 | 9384 | 14045 | 14848 | 11151 | 5653 | 2403 |
| Cost/hospital admission (in US$) | Men | 1904 | 1595 | 1869 | 2094 | 2178 | 2005 | 1061 |
|  | Women | 816 | 1262 | 1504 | 1845 | 2042 | 1516 | 644 |
