# Supplementary material for: Low back pain should be considered a health and research priority in Brazil: Lost productivity and healthcare costs between 2012 to 2016
Source: PLoS One. 2020 Apr 1;15(4):e0230902. doi: 10.1371/journal.pone.0230902 (PMC7112211; doi:10.1371/journal.pone.0230902)
Supplement: S2 Table — Q%: Percentage relative to the total number of procedures reported during outpatient care in each year; C%: Percentage relative to the total costs in each year. (DOCX) [file pone.0230902.s002.docx]

**S2 Table. Overview of clinical and diagnostic procedures/services adopted during outpatient care of individuals with low back pain between 2012-2016. Q%: Percentage relative to the total number of procedures reported during outpatient care in each year; C%: Percentage relative to the total costs in each year.**

| **Clinical procedures** | | |  |  |  |  |  | |
| --- | --- | --- | --- | --- | --- | --- | --- | --- |
| **Year** | **Quantity** | **Total Costs (US$)** | **Most used** | **Quantity** | **Q%** | **Costs (US$)** | **C%** |  |
| **2012** | 6.465.110 | 21.596.399 | Physiotherapy interventions for motor changes | 6.304.270 | *97,5* | 19.692.224 | *91,2* |  |
|  |  |  | Intensive physical rehabilitation | 123.347 | *1,9* | 1.788.559 | *8,3* |  |
|  |  |  | Physiotherapy for neuromusculoskeletal disorders without complications | 33.145 | *0,5* | 103.478 | *0,5* |  |
|  |  |  | *Total* | *6.460.762* | *99,9* | *21.584.262* | *99,9* |  |
| **2013** | 6.106.433 | 19.351.600 | Physiotherapy interventions for motor changes | 5.954.297 | 97,5 | 17.572.399 | 90,8 |  |
|  |  |  | Intensive physical rehabilitation | 123.999 | 2,0 | 1.697.669 | 8,8 |  |
|  |  |  | Physiotherapy for neuromusculoskeletal disorders without complications | 23.257 | 0,4 | 68.553 | 0,4 |  |
|  |  |  | *Total* | *6101553* | *99,9* | *19.338.622* | *99,9* |  |
| **2014** | 6.038.545 | 17.556.660 | Physiotherapy interventions for motor changes | 5.917.532 | 98,0 | 16.418.052 | 93,5 |  |
|  |  |  | Intensive physical rehabilitation | 79.399 | 1,3 | 1.021.593 | 5,8 |  |
|  |  |  | Physiotherapy for neuromusculoskeletal disorders without complications | 19.341 | 0,3 | 53.580 | 0,3 |  |
|  |  |  | *Total* | *6016272* | *99,6* | *17.493.224* | *99,6* |  |
| **2015** | 5.877.717 | 15.659.163 | Physiotherapy interventions for motor changes | 5.720.366 | 97,3 | 14.368.673 | 91,8 |  |
|  |  |  | Intensive physical rehabilitation | 91.813 | 1,6 | 1.067.395 | 6,8 |  |
|  |  |  | Physiotherapy for neuromusculoskeletal disorders without complications | 22.543 | 0,4 | 56.427 | 0,4 |  |
|  |  |  | *Total* | *5834722* | *99,3* | *15.492.496* | *98,9* |  |
| **2016** | 5.739.864 | 14.372.056 | Physiotherapy interventions for motor changes | 5.548.671 | 96,7 | 13.112.005 | 91,2 |  |
|  |  |  | Intensive physical rehabilitation | 86.391 | 1,5 | 944.942 | 6,6 |  |
|  |  |  | Physiotherapy for neuromusculoskeletal disorders without complications | 23.225 | 0,4 | 54.695 | 0,4 |  |
|  |  |  | *Total* | *5658287* | *98,6* | *14.111.643* | *98,2* |  |
| **Total (2012-2016)** | **30.227.669** | **88.535.877** | ***Total clinical (most used)*** | ***30.071.596*** | ***99,5*** | ***88.020.247*** | ***99,4*** |  |
| **Diagnostic procedures** | | |  |  |  |  |  | |
| **Year** | **Quantity** | **Total Costs (US$)** | **Most used*** | **Quantity** | **Q%** | **Costs (US$)** | **C%** |  |
| **2012** | 202.570 | 22.529.984 | CT-scan | 91.707 | 45,3 | 6.121.493 | 27,2 |  |
|  |  |  | Magnetic Resonance Imaging | 84.141 | 41,5 | 15.117.803 | 67,1 |  |
|  |  |  | *Total* | *175.848* | *86,8* | *21.239.296* | *94,3* |  |
| **2013** | 186.312 | 20.250.153 | CT-scan | 83.913 | 45,0 | 5.285.819 | 26,1 |  |
|  |  |  | Magnetic Resonance Imaging | 82.137 | 44,1 | 13.928.323 | 68,8 |  |
|  |  |  | *Total* | *166.050* | *89,1* | *19.214.141* | *94,9* |  |
| **2014** | 203.122 | 20.022.548 | CT-scan | 86461 | 42,6 | 5.112.852 | 25,5 |  |
|  |  |  | Magnetic Resonance Imaging | 85636 | 42,2 | 13.654.318 | 68,2 |  |
|  |  |  | *Total* | *172097* | *84,7* | *18.767.171* | *93,7* |  |
| **2015** | 194.838 | 17.948.633 | CT-scan | 77.002 | 39,5 | 4.149.308 | 23,1 |  |
|  |  |  | Magnetic Resonance Imaging | 87.495 | 44,9 | 12.684.290 | 70,7 |  |
|  |  |  | *Total* | *164497* | *84,4* | *16.833.597* | *93,8* |  |
| **2016** | 187.074 | 16.024.856 | CT-scan | 73.172 | 39,1 | 3.701.270 | 23,1 |  |
|  |  |  | Magnetic Resonance Imaging | 83.826 | 44,8 | 11.438.047 | 71,4 |  |
|  |  |  | *Total* | *156998* | *83,9* | *15.139.317* | *94,5* |  |
| **Total (2012-2016)** | **973.916** | **96.776.175** | ***Total diagnostic (most used)*** | ***835.490*** | ***85,8*** | ***91.193.522*** | ***94,2*** |  |
| * The information refers only to diagnostic imaging adopted in the spine region. | | | | | | | | |
