# Supplementary material for: Low back pain should be considered a health and research priority in Brazil: Lost productivity and healthcare costs between 2012 to 2016
Source: PLoS One. 2020 Apr 1;15(4):e0230902. doi: 10.1371/journal.pone.0230902 (PMC7112211; doi:10.1371/journal.pone.0230902)
Supplement: S3 Table — SE: Standard Error; 95%CI: 95% confidence interval. (DOCX) [file pone.0230902.s003.docx]

**S3 Table: Estimated marginal means on absence from work (in days), and indirect costs (in US$), stratified by gender (male; female), Economic activity (commerce; transports; industry; public servant; rural work), and type of benefit (Work-Related and Non-Work-Related Benefit). SE: Standard Error; 95%CI: 95% confidence interval.**

| **Absence from work**  **(in days)** | Mean (SE) | 95%CI |  | **Lost productivity costs**  **(in US$)** | Mean (SE) | 95%CI |
| --- | --- | --- | --- | --- | --- | --- |
| Gender: |  |  |  | Gender: |  |  |
| Male* | 88.09 (1.98) | 54.23; 91.94 |  | Male* | 2684.6 (74.7) | 2538.2; 2831.1 |
| Female | 84.03 (1.97) | 80.16; 87.89 |  | Female | 2022.3 (74.7) | 1875.8; 2168.8 |
| Type of benefit: |  |  |  | Type of benefit: |  |  |
| Work-Related Benefit† | 87.09 (1.98) | 83.22; 90.97 |  | Work-Related Benefit† | 2332.9 (74.9) | 2186.0; 2479.8 |
| Non-Work-Related Benefit | 85.02 (1.96) | 81.16; 88.87 |  | Non-Work-Related Benefit | 2374.0 (74.6) | 2227.7; 2520.3 |
| Economic activity: |  |  |  | Economic activity: |  |  |
| Transports^1^ | 84.82 (1.22) | 82.42; 87.21 |  | Transports^1^ | 3607.6 (76.2) | 3458.3; 3756.9 |
| Public servant^2^ | 79.83 (5.64) | 68.78; 90.89 |  | Public servant^2^ | 2114.8 (187.7) | 1746.9; 2482.7 |
| Rural work^3^ | 109.39 (0.28) | 108.84; 109.94 |  | Rural work^3^ | 1872.5 (6.6) | 1859.3; 1885.6 |
| Industry^4^ | 69.99 (7.95) | 54.40; 85.58 |  | Industry^4^ | 1870.6 (313.3) | 1256.3; 2484.8 |
| Commerce^5^ | 86.25 (0.11) | 86.03; 86.47 |  | Commerce^5^ | 2301.7 (3.9) | 2294.0; 2309.5 |
| Pairwise comparisons: *Significantly different compared to women (*p*<0.01); † Significantly different compared to social security benefits (*p*<0.01); 1. Significantly different compared to rural work (*p*<0.01); 2. Significantly different compared to rural work (*p*<0.01); 3. Significantly different compared to all others (*p*<0.01); 4. Significantly different compared to rural work (*p*<0.01); 5. Significantly different compared to rural work (*p*<0.01). | | |  | Pairwise comparisons: *Significantly different compared to women (*p*<0.01); † Significantly different compared to social security benefits (*p*<0.01); 1. Significantly different compared to all others (*p*<0.01); 2. Significantly different compared to transports (*p*<0.01); 3. Significantly different compared to transports and commerce (*p*<0.01); 4. Significantly different compared to transports (*p*<0.01); 5. Significantly different compared to rural work and transports (*p*<0.01). | | |
